# Instrument for Evaluation and Training of Decision Making in Dual Tasks in Soccer: Validation and Application

**DOI:** 10.3390/s24216840

**Published:** 2024-10-24

**Authors:** Lucas Romano Oliveira de Souza, Alexandre Luiz Gonçalves de Rezende, Jake do Carmo

**Affiliations:** 1 Federal Institute of Brasília, Brasília 72.220-260, Brazil; 2 Department of Physical Education, University of Brasilia, Brasília 70.910-900, Brazil; rezende@unb.br (A.L.G.d.R.); jake@unb.br (J.d.C.)

**Keywords:** reaction time, soccer, dual-task, sports training, performance evaluation

## Abstract

Training in team sports such as soccer requires advanced technical and tactical skills for effective decision-making, particularly when executing a shot. This study validates an innovative instrument, a training platform (TP), designed to measure and enhance decision-making in dual-task scenarios. The TP aims to improve visual–motor reactions in multitask environments that simulate real game conditions. Equipped with an LED panel, main circuitry, ball sensor, and targets, the TP challenges players to kick the ball in response to the illumination of the final LED array on the panel while hitting a designated target. The study evaluated three parameters: reaction time (RT), ball speed (BS) and accuracy. To validate the TP against a gold standard (GS), we conducted correlation analyses. The results exhibited very strong correlations for both RT (r = 0.997) and BS (r = 0.994). The mean differences between TP and GS measurements were 13 ± 15 ms for RT and 0.1 ± 0.5 km/h for BS. Bland–Altman plots revealed trend lines obtained by a simple linear regression of r = −0.507, *p* = 0.307 for RT and r = 0.134, *p* = 0.077 for BS. The TP effectively simulates game scenarios, offering advantages such as low-cost components, installation flexibility, test variability, instant feedback, and integration of physical and cognitive components of sports performance.

## 1. Introduction

Team sports, like soccer, demand complex technical and tactical skills from players. An effective player must simultaneously monitor teammates and opponents while analyzing possibilities for progression with and without the ball. During a match, players face diverse situations involving rapid interpretation of various stimuli to determine which specific soccer technique to execute (shooting, passing, dribbling or retaining the ball) [1,2,3,4,5]. Analyzing game circumstances offers crucial information for a successful decision-making among multiple options [6].

Research on perception, cognition and action has demonstrated the interconnection of perceptual–cognitive skills in decision-making processes in team sports [5,7,8]. The identification and extraction of information from the environment (perception) is influenced by the ability to direct attention from memory and existing knowledge about the game (cognition), which supports the selection and execution of responses within the player’s capabilities (action) [5,9]. From this perspective, studies aim to understand how players perceive and recognize patterns, detect relevant signals, and identify postural and visual cues as intrinsic and inseparable parts of tactical decision-making during a game [10,11,12,13].

Soccer encompasses complex activities requiring dual cognitive processing that includes both intuitive and reflective responses. In this sense, information processing is classified into two types: (1) intuitive processing and (2) reflective processing. Intuitive processing occurs quickly and autonomously, almost as if triggered by a stimuli. These are examples where decisions are practically automatic due to time constraints on information processing. In contrast, reflective processing relies on a working memory to direct attention, considering both the player’s and opponents’ abilities to analyze circumstances and construct a successful play [3,5,14,15].

Sports training can improve soccer players’ decision-making by analyzing both intuitive and reflective cognitive processing. Intuitive processing enhances quick reactions in high-pressure situations, while reflective processing is essential for strategic planning and analysis. Training involving naturalistic movements—incorporating specific soccer techniques like passing, shooting, or dribbling—reinforces these skills. Realistic game simulations during training sessions help athletes develop the capacity for quick and effective responses to visual and physical stimuli [5,16].

Sports training using sport-specific movements is central in enhancing athlete performance, particularly by improving reaction time (RT) in players across various sports disciplines. Tracking the evolution of a player’s performance in dynamic sports frequently involves evaluating RT [17,18,19]. It is well known that the ability to react, that is, to respond promptly to visual and/or auditory stimuli, can influence the execution of movements [20,21,22,23]. Nonetheless, the current literature lacks equipment that measures RT in an ecological manner, using sport-specific movements. Most studies measure RT through sensors or button touches with limbs or via electromyography, which may not fully capture the complexity of real game situations [18,21,22,24,25].

Currently, performance evaluation instruments measure specific skills in isolation or through a combination of isolated tests. While these instruments, along with direct player observation, are commonly used to monitor the efficiency and effectiveness of a training program, and measure the improvement of sports skills, they may not adequately address the complex characteristics of the game [26,27]. There is a need for instruments that assess tactical skills by simultaneously articulating perceptual-motor components, technical capacities, and at least two decision-making possibilities. Such instruments could better simulate the dynamic situations and random variations inherent in sports, particularly focusing on game-specific motor actions [1,6,28].

Technological advancements are increasingly being integrated into sports training. Several instruments for performance evaluation and skills training in soccer now aim to simulate real game conditions. For example, Igloo Vision Ltd., developed in Shropshire, UK, features an immersive dome installed in a test room where players view real game situations and make passing decisions by kicking a ball towards a player on a screen projection [29].

Rezzil is a virtual reality-based training equipment simulating several game scenarios. Players, inside a test room, wear virtual reality (VR) glasses and perform actions that are monitored by motion sensors installed in their boots and shin guards, without using an actual ball [30].

The Footbonaut Training System is installed in a square room holding 64 targets, eight ball launchers, as well as sound and light markers. Players receive balls from launchers and kick the balls to the marked targets. This system also needs a specially installed room for operation [2].

However, these advanced systems have restrictions. First, they cannot be used in the field as they require controlled laboratory conditions. Second, their high cost limits their use in environments with financial constraints or in sports initiation programs.

Considering the importance of evaluating and training tactical skills in real game situations, we developed an instrument that measures performance based on reaction time, speed and shot accuracy. The equipment allows soccer players to train their skills in dual-task activities, enabling them to read game circumstances accurately (perception), decide on the suitable action (cognition) and execute their shot with precision and agility (action).

The instrument consists of a panel composed of ten LED arrays arranged laterally to the player. These arrays light up in sequence at varying speeds, as programmed. Concurrently, another LED array above the targets in front of the player indicates the target (right or left) to be hit. The timing of this target indication is also programmable. The player is then instructed to kick the ball as quickly as possible when the last LED array lights up, aiming to hit the indicated target. This equipment is wireless, mobile and is made with low-cost components. It offers data for each attempt, including the reaction time of the shot, ball speed, and shot accuracy. The data were recorded on an SD memory card and displayed on the equipment panel for both the evaluator and the player.

## 2. Materials and Methods

### 2.1. Participants

The validation study involved five male volunteers with the following features: height: 1.74 ± 0.12 m; weight: 73.0 ± 15.8 kg; age: 25.8 ± 9.7 years; health status: all healthy, without mobility issues; handedness: four right-handed and one left-handed. Participants were instructed to abstain from caffeine, alcohol and strenuous exercise for 24 h prior to the evaluation sessions. The study adhered to the Declaration of Helsinki guidelines and received approval from the Ethics Committee of UnB—Faculty of Health Sciences of the University of Brasília (approval number 3,580,462). All participants provided informed consent by signing the Free and Informed Consent Form (ICF).

### 2.2. Training Platform System

**Figure 1:**
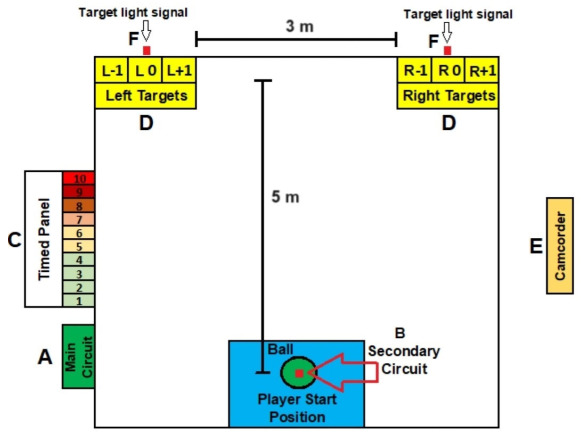
Representation of the training platform system. (A) Main circuit. (B) Secondary circuit. (C) Timed panel, with an indication of the lighting sequence of the 10 time units. (D) Targets, left and right. (E) Camera used for system validation. (F) Target indicator array.

The Training Platform (TP) system consists of a main circuit and a secondary circuit (Figure 1). The main circuit is formed by an Arduino Mega 2560 for data processing and result presentation (A). The secondary circuit consists of an Arduino Nano connected to Bluetooth, which sends ball kick sensor signals to the main circuit (B). The system also includes a timed light panel with ten LED arrays (C), which indicate the moment the ball should be kicked. The switching speed of the panel lights is set by the main circuit. There are two targets positioned laterally, one on the right and one on the left, with three possible hit zones each (D). The distances and placement of the timed panel used in the pilot study can be modified according to the coach’s objectives or to diversify the conditions for evaluating the player’s performance. To validate the instrument, a camcorder was positioned on the right side to record the images (E).

The volunteer stands close to the ball, following the sequential lighting of the timed panel. A target is randomly selected, and when the timed panel lights up the last unit, the volunteer must kick the designated target as quickly as possible.

#### 2.2.1. Main Circuit

The main circuit was developed using an Arduino Mega 2560 Rev3 (Arduino, Ivrea, Italy), prototyping board featuring an ATmega 2560 microcontroller (8-bit AVR architecture). This board utilizes 32 digital ports for communication between the LED panel, sensors, and the secondary circuit. The Arduino Mega 2560 was selected primarily for its four native serial communication ports, of which three are utilized in this system. They were distributed as follows: the first serial port (Serial 0) interfaces with the computer for programming uploads. The second port (Serial 1) connects to the Bluetooth module of the ball sensor, while the third port (Serial 2) links to the Nextion display [31,32].

**Figure 2:**
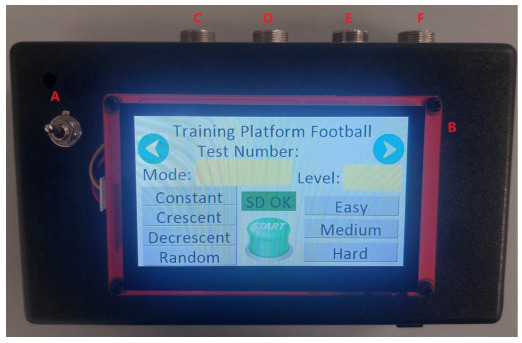
Top view of the main circuit of the soccer training platform. (A) on/off switch. (B) Nextion display. (C) connectors for the right target signals. (D) connectors for the left target signals. (E) power connectors for the timed panel. (F) signal connectors for the timed panel.

A Shield Borne extension for the Arduino Mega 2560 integrates all system components, which are kept in a molded box to guarantee robustness and portability (Figure 2). The Bluetooth module employed is a HC-05 (DSD TECH, Shenzhen, China) in master mode, enabling pairing with the slave module and facilitating serial communication at 9600 bps. The data generated during training sessions were recorded using a micro-SD card module. This module communicates via the SPI interface. To power the Arduino and the Nextion 4,3” model NX4827P043-011C (Nextion, Shenzhen, China) [32] display, which assists the programming interface for the trainer, four 9 V batteries are incorporated into the design.

The data transmission between the main circuit and its peripherals is conducted via cable using Mike Gx16 (Renhotec, Jiangmen, China) circular connectors in order to ensure greater equipment robustness.

#### 2.2.2. Secondary Circuit

The secondary circuit, powered by a 9 V battery, includes an HC-05 Bluetooth module in slave mode. This module communicates with the main circuit, allowing for mobility in its use. In the pilot study, this circuit was positioned five meters from the target line (Figure 1). However, this distance is adjustable based on evaluation or training objectives, ranging from shorter distances up to approximately 15 m while maintaining transmission quality.

During open-field testing, the intense sunlight interfered with the LDR sensor’s ability to detect the moment of ball contact. To address this issue, a black foam receptacle for the ball was created. This receptacle completely blocks external light while permitting unimpeded ball trajectory. The secondary circuit is configured to send a digital signal to the main circuit when the ball’s tangent crosses the line perpendicular to the centrally installed sensor.

#### 2.2.3. Timed Panel

**Figure 3:**
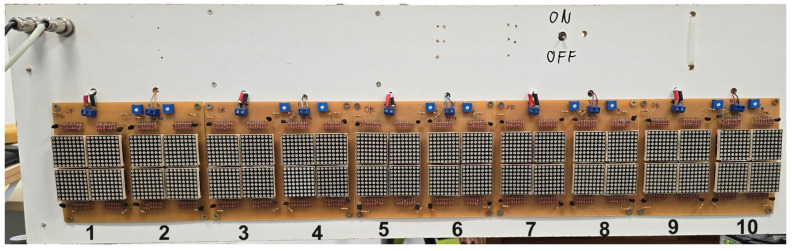
Front view of the timed panel, representing 10 time units. Each unit is composed of four 8 × 8 LED matrices.

The Timed Panel is a light indicator consisting of 10 time units (Figure 3), featuring sequential lighting with varying time intervals. Each unit is formed of four sets of 8 × 8 LED arrays, ensuring good visibility even in bright environments. The Arduino Mega 2560 controls the activation of these cells by sending a 5 V signal through its digital output to a relay, which switches 12 V to the base of a transistor that works as a key in the matrix. A trimpot at the transistor’s base controls the base current, allowing for brightness regulation of the array.

The rear of the panel houses the 16-channel 12 V relay module for driving the LED arrays, the power distribution board, the battery and the 12 V voltage regulator.

#### 2.2.4. Targets

**Figure 4:**
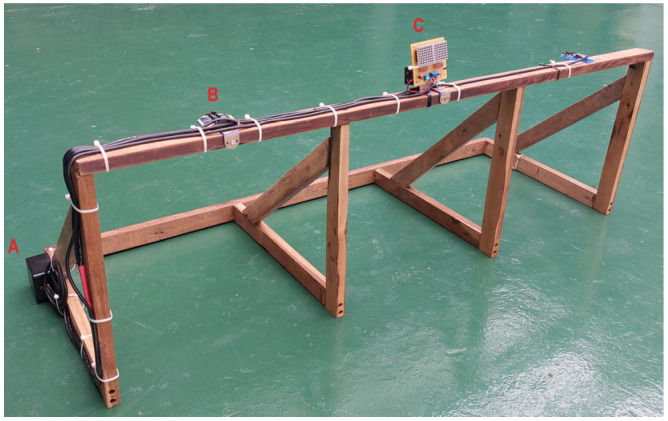
Target assembly. (A) Junction box. (B) Ultrasonic sensors. (C) Target indicator light.

The target system employs three HC-SR04 (FBELE, YinZhou, China) ultrasonic sensor modules [33] strategically positioned on the crossbar, centered on each target (Figure 4). The sensors are calibrated to detect when a ball has fully entered the target area. The “NewPing.h” library was utilized to optimize the sensor’s performance for this specific application. This allowed for the adjustment of the ultrasonic sensor’s measuring range to 30 cm, which is appropriate for the target’s dimensions of 40 cm × 40 cm. A custom connection box was developed to facilitate the interconnection between the main circuit, the target sensors, and the target signal circuit.

#### 2.2.5. Human–Machine Interface

The human–machine interface (HMI) utilizes a Nextion 4.3” capacitive touch screen display. This display was selected for its larger information presentation area, faster processing capabilities, and improved data entry due to its capacitive touch technology. The training platform system features three main screens for user interaction [34].

**Figure 5:**
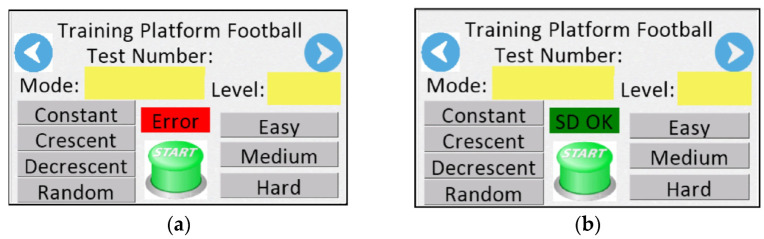
First screen: (**a**) System initialization screen displaying memory card error and training parameter configuration options; (**b**) System initialization screen confirming successful memory card detection and displaying training parameter configuration options.

On the first screen (Figure 5), users can confirm the operation of the micro-SD card, set the mode of the lighting sequence for the timed light panel and choose the training difficulty level. The green button (start) initiates the scheduled training. If the card is misread **(Figure 5**a), the training cannot commence, and the user is prompted to correctly insert the card for a new reading. Upon successful reading, a confirmation message “SD OK” is displayed (Figure 5b).

The lighting speed of the time units on the timed panel can be set to four different sequences:

(1) Constant mode: Each array lights up at a fixed 1000 ms interval.
(2) Crescent mode (accelerated sequence): The second array lights up after 1000 ms, with each subsequent cell illuminating 50 ms faster than the previous one.
(3) Descending mode (decelerated sequence): The second array lights up after 1000 ms, with each subsequent cell illuminating 50 ms slower than the previous one.
(4) Random mode: Initial lightning occurs at 1000 ms, with following illuminations varying randomly by ±300 ms. This range ensures perceptible variation while maintaining a minimum interval of 300 ms and a maximum of 2000 ms to avoid extreme acceleration or deceleration.

Test difficulty levels include easy, medium and hard, determining when the random target is visually indicated to the athlete. In easy mode, the target is indicated at the fifth LED array’s illumination; medium model at the seventh; and hard mode at the ninth. Thus, higher difficulty levels reduce preparation time, demanding greater athlete attention to process two different sources of information.

The second screen allows trainers to enter participant identification details for micro-SD card recording, including player initials, test/training date, playing position, age and the category.

**Figure 6:**
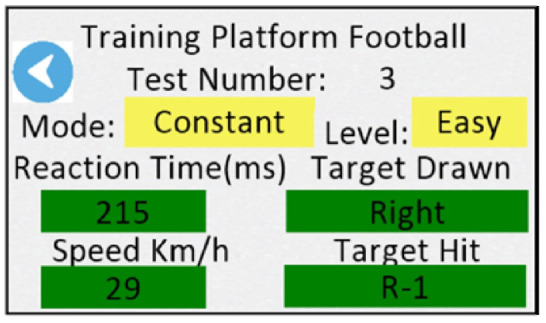
Training results display presenting the details of the test performed, the parameters set for the test and the main results. For example: Test Number: 3. Mode: Constant. Difficulty level: Easy. Reaction time (ms): 215. Ball speed (km/h): 29. Target assigned: Right. Target hit: R-1 (Right, secondary target to the left of the center).

The third screen (Figure 6) displays the most recent shot results:

- Trainer-selected mode and difficulty level;
- Kick reaction time in milliseconds (negative if the kick occurs before the last cell illuminates);
- Estimated ball speed (km/h) based on a 10 m distance between the ball and the target;
- The system randomly selects a target and records the hit. The hit target is indicated as follows: “C0” for a hit on the central target, “L-1” for a hit on the secondary target to the left of the main target, R+1 for a hit on the secondary target to the right of the main target. Here, “C”, “L” and “R” stand for “Center”, “Left” and “Right”, respectively. If the participant does not hit a target, the system automatically waits five seconds to confirm the targets and then displays “Missed target”.

For test number 3, the training platform system was set to constant mode and easy level of difficulty. The system randomly chose the “right” target, and after the participant executed the shot, the main circuit display exhibited the results (Figure 6). The participant hit the “right” target R-1, with a reaction time of 215 ms and a ball speed of 29 km/h.

### 2.3. Protocol

The validation study was conducted in an outdoor setting. Volunteers performed kicks using the training platform following a nine-phase sequential protocol. The test protocol required a minimum of 36 shots. In order to warm up and become familiarized with the training platform, participants performed a five-minute run followed by practice kicks. The practice session used the constant mode at the easy difficulty level (phase 1) until the participant achieved four correct responses in this configuration.

Each phase transition happened after four correct responses. The phase sequence increased in difficulty, as outlined in Table 1. All executed kicks were included in the reaction time analysis. Participants were given two minutes of rest between phases and five minutes of rest after completing every three phases.

**Figure 7:**
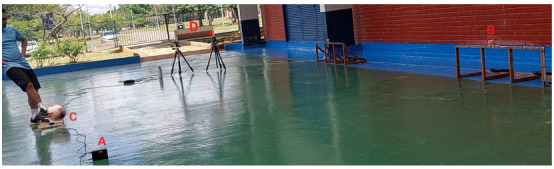
Kick execution on the training platform. See Video S1 in the Supplementary Materials. (A) Kick Signal Circuit. (B) Signal light indicating the ball passing the target. (C) Secondary circuit with ball exit sensor. (D) Timed panel with the last time unit lit, visual indication for the kick.

For each test, the participant was instructed to position themselves near the ball (Figure 7). All kicks were recorded using a Samsung Galaxy A52 smartphone, placed to the right of the kicking platform (A in Figure 1) at a frame rate of 30 Hz.

For the validation of the system, data recorded by the main circuit stored on the micro-SD card were used. The kinematic variables obtained by Kinovea Version 0.9.5 software served as the gold standard (GS) to verify the correct functioning of the equipment [35,36,37]. The same variables—reaction time and ball speed—were extracted from the footage based on the number of frames, which were then transformed into milliseconds. In order to align the measurements collected from both systems, LED indicators were installed to signal the moment of the ball’s departure from the secondary circuit (A in Figure 7) and the ball’s passing the ball over the target (B in Figure 7).

The reaction time obtained by the cinematography was defined as the time from the lighting of the 10th LED array to the lighting of the ball exit signal (A in Figure 7). The ball speed was calculated from the time of the ball’s exit from the secondary circuit (C in Figure 7), indicated by the illumination of the ball exit signal (A in Figure 7) to the time the ball passes through the target sensor, indicated by the ball overtake signal (B in Figure 7). The target distances used to calculate the ball velocity were:

- From the secondary circuit to targets L+1 and R-1: 5.15 m
- From the secondary circuit to targets L 0 and R 0: 5.25 m
- From the secondary circuit to targets L-1 and R+1: 5.44 m

Ball speed was calculated using Equation(1), based on the specific target hit.

(1) $$\mathrm{Ball}\;\mathrm{speed}\;=\frac{\mathrm{target}\;\mathrm{distance}}{\mathrm{target}\;\mathrm{time}}\times 600$$

### 2.4. Statistical Analysis

The Kolmogorov–Smirnov test was employed to verify the normality of the data for the variables of interest (reaction time and ball speed). To validate the TP data against the GS (Cinematography), Spearman correlation tests (r) were conducted on non-parametric data, along with simple linear regression and Bland–Altman analyses. A two-way repeated measures ANOVA was conducted to evaluate the interactions between level and mode, with the Bonferroni test used for post hoc analysis. The magnitudes of the validity correlations were interpreted as follows: very strong (0.90 to 1.00), strong (0.70 to 0.89), moderate (0.50 to 0.69), weak (0.30 to 0.49), and negligible (0.00 to 0.29) [38]. The *p*-value ≤ 0.05 was indicated as a degree of significance. The means and standard deviations of reaction time and measured speed were also analyzed, determining a 95% confidence interval (CI). From the sample mean, the standard error of the mean (SEM), and the margin of error, we presented the average value with its interval for each variable and for the difference between the systems. All statistical calculations were implemented using SPSS version 25 and Microsoft 365 Excel.

## 3. Results

In total, 329 valid kicks were recorded during the testing phase. After data cleansing, which involved removing outliers with reaction times greater than ±2000 ms, we had 325 measurements for reaction time and 176 for ball speed. This difference was due to the fact that only shots that hit the targets were subject to ball speed measurement. Overall, 53% of the shots hit the targets. Table 2 presents the total number of shots per difficulty level, the total number of hits and the percentage of shots that hit the targets.

To extract the values of each variable from the video (reaction time, ball speed and hits on targets), the Kinovea software was used [35]. An experienced operator analyzed each volunteer’s data, taking nearly 1 h and 30 min per volunteer. With five volunteers, the total analysis time was 7 h and 30 min. The quick feedback provided by the proposed instrument is an important advantage over video analysis.

**Figure 8:**
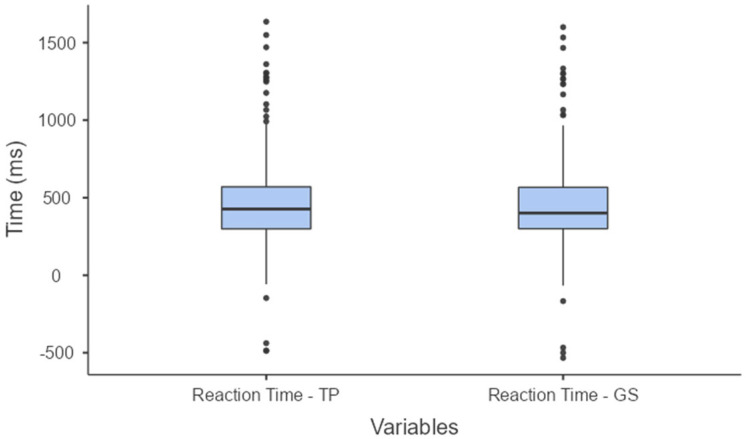
Boxplot comparing the means of the reaction time measurements obtained by the training platform (TP) and gold standard (GS).

The mean value and standard deviation of the TP reaction time measurements was 448 ± 288 ms and the GS was 434 ± 289 ms with a 95% confidence interval (CI), the reaction time (RT) for the training platform (TP) ranged from 425 to 470 ms and for the gold standard (GS) it was 434 ± 289 ms, the RT for GS ranged from 411 to 457 ms, with a 95% CI. (Figure 8). The data showed a non-parametric distribution and a very strong correlation (r = 0.997). The mean difference in the measured values between the two systems was 13 ± 15 ms with a 95% CI, TR (TP vs. GS) ranged from 12 to 15 ms. As shown in Figure 8, the outliers identified in the TP boxplot were also detected in the GS data. These outliers are characteristic of the sample, reflecting variations in the volunteers’ reaction times as detected by both systems.

**Figure 9:**
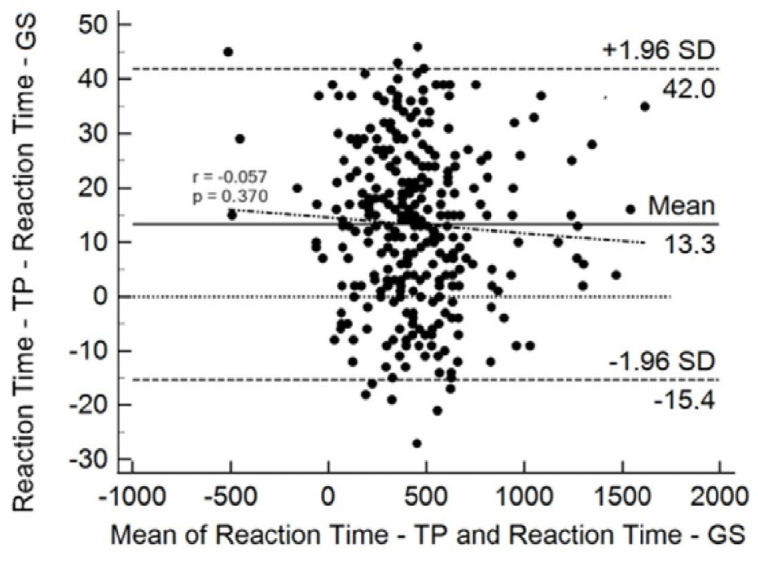
Bland–Altman graph of reaction time measured by training platform (TP) and gold standard (PO).

Figure 9 presents the Bland–Altman graph for the reaction time variable measured by PT and GS. The graph depicts the limits of 1.96 standard deviations, with an upper limit of 42 and a lower limit of -15.4. The trend line obtained by simple linear regression of means and differences (r = −0.507, *p* = 0.307) was included. No heteroscedasticity was observed, indicating that there was non-uniformity of error between the system under test and the GS. This confirms that comparisons between measurement methods are valid and reliable across the entire range of values between samples.

**Figure 10:**
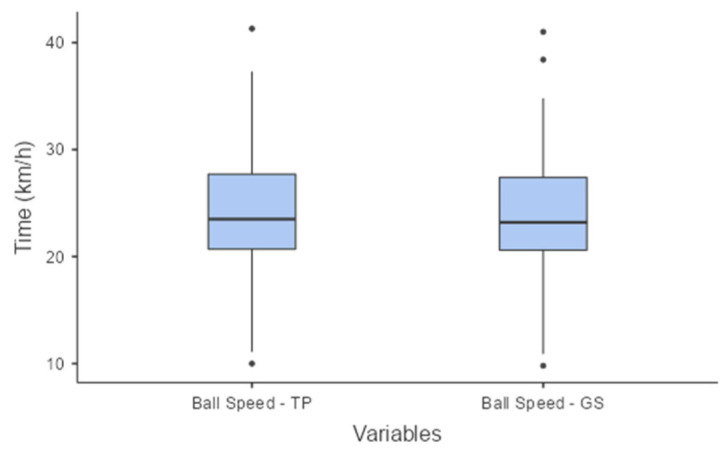
Boxplot comparing the average ball speed measurements obtained by the training platform (TP) and gold standard (GS).

The average ball speed and standard deviation, calculated by the platform, were 23.8 ± 5.7 km/h, with a 95% (CI), the BS for the TP ranged from 23.3 to 24.2 km/h and for the GS it was 23.6 ± 5.6 km/h, the BS for GS ranged from 23.2 to 24.1 km/h, with a 95% CI. (Figure 10). This represents a very strong non-parametric distribution and correlation (r = 0.994). The mean difference in measurements between the two systems was 0.2 ± 0.5 km/h with an IC 95% and the BS (TP vs. GS) ranged from 0.1 to 0.2. In Figure 10, it may be observed that the outliers detected in the boxplot were also identified by the GS. These outliers are typical of the samples due to variations in ball speed after the shot, as perceived by both systems.

**Figure 11:**
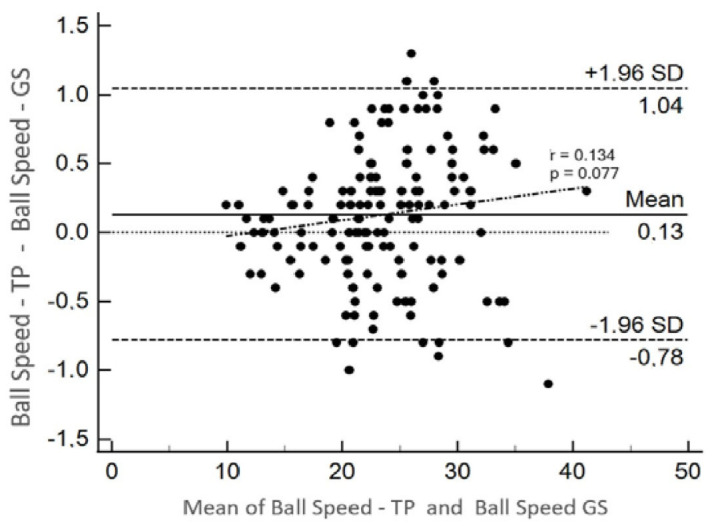
Bland–Altman graph of ball speed measured by the training platform (TP) and gold standard (PO).

Conversely, Figure 11 presents the Bland–Altman graph for the ball speed measured by the two systems, exhibiting an upper limit of 1.04 and a lower limit of −0.78. The trend line was drawn using simple linear regression of the means and differences (r = 0.134, *p* = 0.077). No heteroscedasticity, or non-uniformity of error, was observed between the system under test and the GS, ensuring that comparisons between measurement methods are valid and reliable across the range of values between samples.

Reaction time rises with the difficulty level, as shown in Table 3. For ball speed, there is a slight increase in the average value from easy to medium difficulty levels, which remains stable between the medium and hard levels. The variations observed in the measurements of both variables were detected by both systems.

With variations in the lighting modes of the timed panel, the reaction time presents increasing values as the task complexity increases (constant, descending, increasing and random), as shown in Table 4. There is a small increase in the average value from easy to medium difficulty levels and it remains stable between the medium and hard levels. Both systems detected the variations in the measurements of the two variables.

## 4. Discussion

The main objective of the study was to validate an instrument for evaluating and training decision-making in dual tasks in soccer, based on measures of reaction time, ball speed, and shooting accuracy. This instrument simulates a game action in a context with two sources of visual information. The system under test extracts variables immediately, displays them on the screen, and stores them on a micro-SD card. To validate the system, video data were used as the GS. The extraction of information by the GS took a total of 7 h and 30 min for the five volunteers. One of the advantages of the TP is that it provides immediate feedback.

The TP identified all shots where the ball hit the crossbar, which is where the target sensor was positioned. As seen in Table 2, a 53% hit rate was achieved, with 176 out of 329 shots hitting the targets. The shot accuracy decreased from 60% at the easy difficulty level to 52% at the medium level, with a slight increase to 54% at the hard level. The participants reported not noticing much difference in test execution between the medium and hard difficulty levels; however, this subjective perception requires further investigation. Changing the complexity of the LED panel lighting mode resulted in a decrease in accuracy: constant to 57%, decreasing to 55%, increasing to 47% and a slight increase for random to 54%. The hypothesis for the increase in the random mode is that the average lighting time approaches that of the constant mode, although this must be confirmed with a specific instigation.

The TP had high efficiency in analyzing players’ RTs, exhibiting a very strong correlation (r = 0.997) with the GS system results. The mean difference between the two systems was 13 ± 15 ms. We found that most humans perceive events lasting around 13 ms, so the difference between the systems’ measurements is not noticeable to the naked eye [39] As shown in the boxplot in Figure 8, the TP revealed variations in volunteers’ RT similarly to the GS system.

Table 3 presents the RT values from the TP at the easy (328 ± 240 ms), medium (391 ±165 ms) and hard (516 ± 334 ms) difficulty levels, and from the GS (312 ± 240 ms, 377 ± 166 ms and 506 ± 334 ms), at the same respective levels. Both systems show the same ascending order of mean RT values across difficulty levels. The average RT increases directly proportional to the difficulty level. The standard deviation showed equal values between the systems when compared at the same level. The smallest difference recorded between the systems was 9 ± 15 ms (hard) and the largest was 16 ± 14 ms (easy), demonstrating small systematic and random errors, and indicating strong agreement between the methods.

Table 4 shows an increasing and sequential variation in the RT values obtained from the volunteers for the LED panel lighting modes in the following order: constant, descending, ascending and random. Using the TP, the lowest RT value is 362 ± 147 ms (constant) and the highest is 505 ± 304 ms (random). In the GS, the lowest is 344 ± 152 ms (constant) and the highest is 495 ± 302 ms (random). Both systems verified the same ascending order of average RT values for LED panel lighting modes. The minimum difference recorded between the systems was 9 ± 12 ms (crescent) and the maximum was 13 ± 17 ms (random), exhibiting small systematic and random errors, and indicating strong agreement between the methods.

In both situations, whether involving varying difficulty levels or different LED panel lighting modes, the increase in RT can be attributed to alterations in complexity or the degree of attention required from the participant. Factors such as task uncertainty and complexity affect movement preparation and execution, requiring more complex cognitive processing [40].

The Bland–Altman graph for RT results (Figure 9), shows a uniform distribution of error. Correlating the differences by the means, the trend line (r = −0.057) was verified, and linear regression confirmed the significance of this relationship (*p* = 0.307). The graph indicates that the system under test agrees with the GS in measuring RT.

For ball speed (BS), a very strong correlation (r = 0.994) was also found between the TP system and the GS system. The mean difference in measurements between the two systems was 0.2 ± 0.5 km/h. Most commercial radar-type equipment for measuring ball speed has a measurement resolution of around 1.6 km/h, such as the Bushnell Speedster III, Stalker Pro II+ and Sports Radar Speed Gun SR3600 [41,42]. Comparing the boxplots (Figure 8 and Figure 10) between the two systems showed that the TP was able to detect BS variations in the participants’ kicks.

Table 3 presents the BS values from the TP at easy (22.5 ± 5.4), medium (23.5 ± 4.8) and hard (24.4 ± 6.5) difficulty levels, and from the GS (22.3 ± 5.2, 23.4 ± 4.8, and 24.2 ± 6.4 km/h) at the same respective levels. The mean BS value increases between the easy and medium levels and stabilizes between the medium and hard levels. The standard deviation remains very close between the systems when compared at the same level. The smallest difference recorded between the systems was 0.1 ± 0.4 km/h (hard) and the largest was 0.2 ± 0.5 km/h (easy), demonstrating small systematic and random errors, and suggesting strong agreement between the methods.

Table 4 also shows an increasing and sequential variation in the participants’ BS values for LED panel lighting modes in the following order: constant, descending and ascending, with a slight reduction in random mode. In the TP, the lowest BS value was 23.8 ± 5.7 km/h (constant) and the highest was 23.8 ± 5.9 km/h (increasing). In the GS, the lowest was 23.0 ± 5.2 km/h (crescent) and the highest was 24.1 ± 5.9 km/h (random). Both systems verified the same ascending order of average BS values for LED panel lighting modes. The minimum difference recorded between the systems was 0.1 ± 0.4 km/h (increasing and random) and the maximum was 0.2 ± 0.5 km/h (constant and decreasing), demonstrating small systematic and random errors, and suggesting strong agreement between the methods.

The Bland–Altman graph for BS (Figure 11) shows a uniform distribution of error. Correlating the differences by the means, the trend line (r = 0.134) was verified, and linear regression confirmed the significance of this relationship (*p* = 0.077). The graph indicates that the system under test agrees with the GS in measuring BS.

We conducted a two-way repeated measures ANOVA to evaluate statistically significant differences in the variable’s reaction time and ball speed across the levels of the training platform. Since the easy level was used solely for participant familiarization, we only included the medium and hard levels in this analysis. We found a two-way interaction between level*mode, F(3, 168) = 12.8449, *p* < 0.001, η^2^ = 0.1866. The Bonferroni post hoc analyses revealed statistically significant differences between the means of medium-crescent vs. hard-crescent. For ball speed, there was no statistically significant interaction between level and mode. We performed a Friedman ANOVA (non-parametric test) due to the small sample size for ball speed.

The Bonferroni post hoc analyses showed statistically significant differences between levels, specifically between medium-crescent vs. hard-crescent, and for modes, there were differences between hard-constant vs. hard-crescent, hard-decreasing vs. hard-crescent and hard-crescent vs. hard-crescent; at the medium level, no differences were found.

In short, the training platform demonstrated a strong ability to evalulate reaction time, ball speed and shot accuracy with good precision. Additionally, using the platform in a training setting provides instant feedback, allowing real-time adjustments during training sessions. This advantage can lead to faster and more efficient improvements in player performance. The stored data also allow for generating a history of the participant’s performance over time.

In this study, auditory stimuli were not initially considered. However, the activation of LED cells by relays produced an inadvertent sound effect, which participants reported using to count time. The impact of these additional auditory cues warrants investigation in future studies. Research by Hülsdünker et al. (2021) and Sors et al. (2018) indicates that multisensory stimuli, both visual and auditory, can influence reaction times [24,43].

The strong agreement between the two systems demonstrates that the TP technology is a valid alternative for performance analysis in both training and research contexts. Consistency of data across systems is critical, as the reliability of performance measures is fundamental in the development of effective training protocols. The ability to reproduce results in various training situations allows coaches greater flexibility and accuracy in evaluating athletes’ performance.

The TP offers a wide variety of LED timer panel lighting modes and difficulty levels for target presentation. These parameters can be adjusted in real-time by the coach, facilitating adaptation to each athlete’s specific needs or training session requirements. This flexibility ensures the platform’s utility in diverse training and evaluation scenarios. Each lighting mode applies different cognitive stress to the player, simulating various game situations. This adaptability is particularly advantageous in a dynamic sporting environment where demands and conditions can change rapidly.

The majority of the studies assessing soccer players’ performance related to reaction time or decision-making utilize video game situations, non-sport-specific motor actions, or even electroencephalography and magnetic resonance imaging [7,29,43]. The evaluation conducted with the criterion system (gold standard) yields highly precise values. However, it involves extensive time for video processing, a trained specialist, and a computer with substantial graphics processing capacity. In contrast, the proposed system (TP) provides all data instantly after test completion.

Different equipment such as Igloo Vision Ltd., Rezzil and Footbonaut require specialized facilities and are not easily transportable However, the TP distinguishes itself through its high mobility, variety of test types and applicability in different environments. It can be readily used from laboratories to outdoor training camps. This feature guarantees that players can train in conditions more closely simulating real game scenarios, which is essential for transferring skills learned in training to competitive situations [29,30,44].

The use of robust and more accessible technologies such as ultrasonic sensors, human–machine interfaces, Arduino, Bluetooth and LED panels represents a promising bridge between traditional training and more modern approaches, integrating instant feedback and objective data. Furthermore, the TP’s ease of use, portability and training variability make it a valuable tool for clubs, compared to other training technologies that often have high acquisition and operating costs. In this sense, the TP stands out for its economical and robust design. This technology presents itself as a viable solution for clubs with limited budgets or sports initiation programs, democratizing access to high-quality training tools.

The primary aim of this study was to validate the system by comparing it to an already reliable and recognized system. Future studies will focus on the potential benefits brought by the training system. Training procedures that require participants to perform sport-specific motor actions under task constraints similar to those found on the field have shown better results compared to training that requires simplified responses in less representative conditions [45]. Moreover, the extensive capabilities of the TP system allow us to verify the accuracy of the participants using the dominant and non-dominant lower limbs across various situations.

In summary, this study offers initial evidence supporting the use of the training platform as an effective tool for measuring player performance variables. The practical implications of this study are significant, suggesting that coaches may consider incorporating such technologies to enhance reaction training, peripheral vision, and kicking speed. The equipment can be developed with low-cost components and is easy to install and can be used both on the field and in laboratory settings. Additionally, it can be used for training perceptual-cognitive skills under the sports vision training approach, based on naturalistic sports training to improve sports performance through dual tasks with sport-specific activities. The TP’s ability to integrate dual-tasks—simultaneously requiring motor and decision-making skills—is a substantial advantage over equipment that assesses these skills in isolation. This integration is crucial for developing decision-making skills under pressure; a key competency for success in soccer and other team sports [44,46,47].

Limiting factors of this study include a low number of participants who are of the same gender, similar ages, and skill level, thus belonging to a specific demographic group; however, we emphasize that the sample size and characteristics were sufficient for us to carry out the equipment validation studies.

## 5. Conclusions

Recognizing the importance of evaluating and training tactical skills in real game situations, we developed a system that evaluates performance based on reaction time, shot speed and accuracy. The equipment enables soccer players to train their skills in dual-task activities, integrating the ability to: (a) adequately read game circumstances (perception), (b) make decisions about actions to be performed (cognition), and (c) execute precise and agile shots (action).

The development and implementation of the TP represents a significant advance in training and assessment methodology for team sports, particularly soccer. This study, bridging engineering and sports sciences, leads to the construction of an affordable, easy-to-install instrument capable of reproducing game situations with multiple perceptual-motor tasks. The instrument requires players to monitor dual visual information from two different sources in order to decide when and where to kick the ball to hit a small target.

The instrument can be used both as a test to measure players’ performance as well as a training resource to improve their tactical skills. The collected data can support longitudinal studies on skill development, training adaptations, and the effectiveness of various training methodologies. Evaluating players’ tactical performance helps coaches refine training programs according to each player’s individual characteristics. Additionally, repeatedly exposing players to complex game situations helps to develop the ability to adjust and construct the most suitable responses to game challenges.

The study indicates that the developed TP is capable of simulating distinct game scenarios and offers a controlled environment for training the articulation between perception, decision-making, and action. This equipment has the advantage of being usable on football fields, in laboratories, or in open areas. There is no need for pre-prepared rooms with complex installations. Setting up the platform requires only a few minutes to ensure the correct distances between accessories.

The instrument is composed of easily found low-cost components and can be applied by both sports initiation teachers and competitive team coaches. It is worth for identifying sports talent and as a resource for performance appraisal and skills training.

## Figures and Tables

**Table 1:** Phase progression table, indicating the mode and level sequence.

Phases	Mode	Difficulty Level	Hits
Phase 1	Constant	Easy	4
Phase 2	Constant	Medium	4
Phase 3	Constant	Hard	4
Phase 4	Random	Medium	4
Phase 5	Random	Hard	4
Phase 6	Descending	Medium	4
Phase 7	Crescent	Medium	4
Phase 8	Descending	Hard	4
Phase 9	Crescent	Hard	4

**Table 2:** Total number of shots, hits made by difficulty level and timed panel illumination sequence.

Difficulty Level	Kicks	Hits	Hits %
Easy	42	25	60%
Medium	145	75	52%
Hard	142	76	54%
Constant	107	61	57%
Decrescent	69	38	55%
Crescent	77	36	47%
Random	76	41	54%

**Table 3:** Means ± standard deviations for comparison of reaction times (ms) and kick speed (km/h) at different difficulty levels.

Variable		Level	
	Easy	Medium	Hard
	Mean ± SD	Mean ± SD	Mean ± SD
Reaction time—TP ^1^ (ms)	362 ± 147	395 ± 253	505 ± 304
Reaction time—GS ^2^ (ms)	344 ± 152	383 ±256	495 ± 302
Reaction time—Difference (ms)	18 ± 19	11 ± 15	11 ± 14
Ball speed—TP ^1^ (km/h)	22.5 ± 5.4	23.5 ± 4.8	24.4 ± 6.5
Ball speed—GS ^2^ (km/h)	22.3 ± 5.2	23.4 ± 4.8	24.2 ± 6.4
Ball speed—Difference (km/h)	0.2 ± 0.4	0.1 ± 0.5	0.1 ± 0.4

^1^ Training platform, ^2^ Gold standard.

**Table 4:** Averages ± standard deviations for comparison of reaction times (ms) and ball speed (km/h) in the different lighting modes of the LED panel.

Variable	Level			
	Constant	Decrescent	Crescent	Random
	Mean ± SD	Mean ± SD	Mean ± SD	Mean ± SD
Reaction time—TP ^1^ (ms)	374 ± 239	406 ± 171	509 ± 386	502 ± 233
Reaction time—GS ^2^ (ms)	361 ± 244	393 ± 171	500 ± 395	490 ± 236
Reaction time—Difference (ms)	13 ± 16	13 ± 14	9 ± 12	13 ±17
Ball speed—TP ^1^ (km/h)	23.8 ± 5.7	23.8 ± 5.9	23.3 ± 5.2	24.2 ± 5.8
Ball speed—GS ^2^ (km/h)	23.7 ± 5.5	23.6 ± 5.8	23.0 ± 5.2	24.1 ± 5.9
Ball speed—Difference (km/h)	0.2 ±0.5	0.2 ± 0.5	0.1 ± 0.4	0.1 ± 0.4

^1^ Training platform, ^2^ Gold standard.

## Data Availability

The data that support the findings of this study are available from the corresponding author, L.R.O.d.S., upon reasonable request.

## Supplementary Materials

The following supporting information can be downloaded at: https://github.com/LRomano21/LRomano21 (accessed on 21 October 2024), Video S1: Demonstration of the operation of the soccer training platform. Kick execution in crescent mode configuration, hard level, target selected R-1, and target hit R-1.
